# Supplementary material for: The Ecophysiological Performance and Traits of Genera within the Stichococcus-like Clade (Trebouxiophyceae) under Matric and Osmotic Stress
Source: Microorganisms. 2021 Aug 26;9(9):1816. doi: 10.3390/microorganisms9091816 (PMC8472729; doi:10.3390/microorganisms9091816)
Supplement: Supplementary file 1 [file microorganisms-09-01816-s001.zip › P2_Tab.S1_HPLC_abs_values.pdf]

		0 S_A			30 S_A		
**Organism**	**Strain**	**Sorbitol**	**Sucrose**	**Total**	**Sorbitol**	**Sucrose**	**Total**
Desmococcus olivaceus	SAG 1.92	322	28	349	48	-	48*
Deuterostichococcus marinus	J1303	116	106	222	93	135	228
Deuterostichococcus tetrallantoideus	ASIB-IB-37	28	44	72	89	45	134
Diplosphaera epiphytica	SAG 11.88	376	69	445	200	57	257*
Protostichococcus edaphicus	SAG 2481	230	74	303	331	34	365
Pseudostichococcus monallantoides	SAG 380-1	89	9	97	240	18	258
Stichococcus sequoieti	LB 1820	242	111	353	120	74	362
Stichococcus undulatus	CALU-1142	295	126	421	311	57	285*
Stichococcus bacillaris	CCAP 379/1A	151	121	273	288	33	153*
Stichococcus bacillaris	SAG 56.91	200	127	327	228	56	367
Tetratostichococcus jenerensis	J1302	74	17	91	129	34	163
Tritostichococcus solitus	SAG 2406	81	18	99	215	12	227

Tab. S1. Absolute concentrations of sorbitol and sucrose measured in cellular extracts of the strains after growth in 0 S_A and 30 S_A via HPLC analysis. $\text{Totals}_{30\ SA}$ marked with a "*" indicate a net decrease in osmolyte concentration compared to $\text{Totals}_{0\ SA}$. Values are expressed in $\mu mol \cdot g^{-1}$ algal dry weight.
