# Supplementary material for: The Ecophysiological Performance and Traits of Genera within the Stichococcus-like Clade (Trebouxiophyceae) under Matric and Osmotic Stress
Source: Microorganisms. 2021 Aug 26;9(9):1816. doi: 10.3390/microorganisms9091816 (PMC8472729; doi:10.3390/microorganisms9091816)
Supplement: Supplementary file 1 [file microorganisms-09-01816-s001.zip › P2_Tab.S2_PAM_abs_values.pdf]

Strain ID	Taxon identity	Initial avg.	Final avg.	% Initial, 24 hrs
ASIB-IB-37	*Deuterostichococcus tetrallantoideus*	0.54 ± 0.03	0.20 ± 0.34	36.64
J1303	*Deuterstichococcus deasonii*	0.49 ± 0.04	0.27 ± 0.28	54.79
SAG 11.88	*Diplosphaera epiphytica*	0.51 ± 0.00	0.24 ± 0.25	47.28
SAG 2481	*Protostichococcus edaphicus*	0.38 ± 0.05	0.16 ± 0.20	42.57
SAG 380-1	*Pseudostichococcus monallantoides*	0.52 ± 0.02	0.28 ± 0.23	53.03
CALU 1142	*Pseudostichococcus undulatus*	0.57 ± 0.02	0.17 ± 0.13	29.92
LB 1820	*Pseudostichococcus sequoieti*	0.55 ± 0.02	0.24 ± 0.27	43.07
CCAP 379/1A	*Stichococcus bacillaris*	0.46 ± 0.04	0.25 ± 0.20	53.38
SAG 56.91	*Stichococcus bacillaris*	0.49 ± 0.01	0.39 ± 0.25	78.31
J1302	*Tetratostichococcus jenerensis*	0.40 ± 0.02	0.18 ± 0.12	46.43
SAG 2406	*Tritostichococcus solitus*	0.55 ± 0.02	0.20 ± 0.30	35.90

Tab. S2. The average recovery of Y(II) values at 24 hours per strain, expressed as a percentage of initial Y(II). Averages over three experimental replicates per strain were taken. Higher recovery percentages indicate less damage to the photosynthetic apparatus.
