# Supplementary material for: The Ecophysiological Performance and Traits of Genera within the Stichococcus-like Clade (Trebouxiophyceae) under Matric and Osmotic Stress
Source: Microorganisms. 2021 Aug 26;9(9):1816. doi: 10.3390/microorganisms9091816 (PMC8472729; doi:10.3390/microorganisms9091816)
Supplement: Supplementary file 1 [file microorganisms-09-01816-s001.zip › Table S3.pdf]

| **Strain** | **mg · L$^{-1}$** |
| --- | --- |
| J1303 *Deuterstichococcus marinus* | 22.27 ± 12.00 |
| ASIB-IB-37 *Deuterostichococcus tetrallantoideus* | 26.69 ± 17.74 |
| SAG 11.88 *Diplosphaera epiphytica* | 28.22 ± 3.640 |
| SAG 2481 *Protostichococcus edaphicus* | 8.793 ± 3.246 |
| SAG 380-1 *Pseudostichococcus monallantoides* | 5.526 ± 1.638 |
| LB 1820 *Pseudostichocococcus sequoieti* | 14.28 ± 9.455 |
| CALU-1142 *Pseudostichococcus undulatus* | 25.63 ± 8.925 |
| CCAP 379/1 *Stichococcus bacillaris* | 25.16 ± 13.21 |
| SAG 56.91 *Stichococcus bacillaris* | 37.51 ± 8.627 |
| J1302 *Tetratostichococcus jenerensis* | 17.65 ± 10.96 |
| SAG 2406 *Tritostichococcus solitus* | 5.480 ± 4.585 |

**Tab. S3.** The average Chl*a* concentrations of 200 µL biomass dropped onto the filters of the dehydration experiment, expressed as mg Chl*a* · L$^{-1}$ ($n$ = 3 per strain).
